# Biliary Leakage Following Pancreatoduodenectomy: Experience from a High-Volume Center

**DOI:** 10.1089/pancan.2021.0014

**Published:** 2021-12-24

**Authors:** Waqas Farooqui, Luit Penninga, Stefan Kobbelgaard Burgdorf, Jan Henrik Storkholm, Carsten Palnæs Hansen

**Affiliations:** Department of Surgery, Rigshospitalet, Copenhagen University Hospital, Copenhagen, Denmark.

**Keywords:** pancreatoduodenectomy, complications, biliary fistula, postoperative complication, biliary leak, high-volume center

## Abstract

**Purpose:** Hepaticojejunostomy leak and bile fistula after pancreaticoduodenectomy (PD) are less frequent than pancreatic leaks. Patients with biliary fistula (BF) have an increased risk of serious complications and an extended hospital stay. This study has investigated the occurrence and outcome of BF.

**Methods:** All patients who underwent a PD from January 1st, 2015 to December 31st, 2019 were included. The significance of multiple risk factors was examined. Univariate analysis was used to identify predictive variables for postoperative BF.

**Results:** Of the 552 patients who underwent PD, 38 patients (6.7%) developed a BF. Patients with nonmalignant diagnoses and malignancies without bile duct obstruction had a greater risk of developing BF. BF did not increase the mortality, though most patients had complications, including surgical site infections, intraabdominal abscesses, and an extended hospital stay.

**Conclusion:** BF after PD leads to an increased risk of subsequent complications and an extended hospital stay but does not increase mortality. Patients with nonmalignant diagnoses and malignancies without bile duct obstruction have an increased risk of BF.

## Introduction

Biliary fistula (BF) after pancreaticoduodenectomy (PD) is reported with an incidence of up to 8%.^1^ BF occurs less frequently than postoperative pancreatic fistulas (POPF) and has a lower rate of complications and mortality.^2,3^ Thus, BF has caught less attention compared with POPF. However, the development of BF increases the risk of sequelae such as intraabdominal abscesses and biliary peritonitis with an extended hospital stay and increased mortality.^4^ Thus, more attention must be given to BF and ways to reduce prevalence.

Excessive skeletonization of the hepatic duct, a small duct diameter, as well as anastomosis to the common bile duct (CBD) rather than the common hepatic duct (CHD) are among the known risk factors.^4–6^ Reconstruction techniques, percutaneous biliary drainage, and intraoperative T-tube placement are among many attempted protective measures with little success.^4,7^ Preoperative prediction of BF could be advantageous, allowing for early management, but has not been thoroughly assessed for PD.^8^

According to the International Study Group of Liver Surgery (ISGLS), biliary leakage is defined as a measured bilirubin level in drain fluid three times above bilirubin levels in plasma, on or after the third postoperative day (POD). BF is then classified according to the severity of the leak. Grade A defines a BF without noteworthy deviations from a standard postoperative regime. With grade B there is a need for additional radiological and pharmacological intervention and, in grade C there is a need for surgical intervention.^9^ Not all BF need intervention, as up to half of the identified biliary leaks resolve spontaneously.^10^

This study from a single high-volume center aimed at investigating the incidence of BF, identifying risk factors, and describing the subsequent complications of BF after PD.

## Methods

### Patient data

The study included all 552 patients who underwent a PD at our institution from January 1st, 2015 to December 31st, 2019. Only BF categorized as Grade B and C were included in the statistical analysis, as they result in changes in the postoperative course. All patients were included regardless of the surgical indication. Patient data were extracted from the electronic patient journal system EPIC (Epic Systems Corporation, Wisconsin, USA) and the department's prospectively maintained database of pancreatic operations.

### Surgery and postoperative care

All patients were operated on by four specialized hepatobiliary pancreatic surgeons. PD was performed *en bloc* with prepyloric amputation, resection of the CHD below the confluence of the right and left hepatic duct, and resection of the pancreatic head, duodenum, and gall bladder. The CHD was divided with a knife. Reconstruction was performed by retrocolic pancreatojejunostomy, hepaticojejunostomy, and antecolic gastrojejunostomy, with all anastomoses on the same segment of the small bowel and a distance from the hepaticojejunostomy to the gastrojejunostomy of at least 40 cm.

The anastomosis between the pancreas and the jejunum was performed either as an invaginated anastomosis between the small intestine and the pancreas^11^ or as a duct-to-mucosa procedure^12^ depending on the texture and the calibration of the major pancreatic duct. The anastomosis between the hepatic duct and the jejunum was performed as an end-to-side single-layer procedure with long-term resorbable, monofilament sutures and placed 10 cm from the pancreatojejunostomy.

In the case of dilated ducts, running sutures, size 4-0, were primarily used, and in the case of non-dilated ducts, interrupted sutures, size 5-0, were used with the knots tied on the outside to keep the lumen in the anastomosis as wide as possible. A single surgical drain was placed intraoperatively, behind the hepaticojejunostomy and pancreaticojejunostomy.

Measurements of bile duct size were not done intraoperatively, but its calibration was radiologically defined preoperatively by computed tomography (CT) scan or magnetic resonance imaging scan, as either dilated or non-dilated/small calibrated.

A standard regime for postoperative care included mobilization and physiotherapy from POD 1, early removal of nasogastric tube, and oral feeding. Intravenous antibiotics, predominantly cefuroxime (Fresenius Kabi, Denmark), and metronidazole (B. Brain Medical, Denmark) were administered during the first three PODs. Preoperative epidural catheters were kept until POD 4, and the intraoperatively placed drain was removed on POD 5 except in the case of BF or POPF. If bile was recorded in the abdominal drain, a percutaneous transhepatic cholangiography (PTC) was carried out to confirm the leak and during the same procedure external biliary drainage to drain the bile duct and to avoid further intraabdominal accumulation of bile.

Moreover, a CT scan of the abdomen was commonly performed to visualize any additional drainable fluid accumulations, in case of which an ultrasonographically guided drainage was performed. Supportive measures were initiated once BF was confirmed and included fluid therapy and antibiotics according to culture. Intensive care unit (ICU) admission or re-operation was only undertaken in case of clinical deterioration and onset of organ failure.

### Endpoints

Baseline characteristics for each patient included patient age, gender, body mass index (BMI), and the American Society of Anesthesiologists (ASA) Physical Status Classification.^13^ Primary endpoints included the occurrence of BF and related complications, 30 and 90 days mortality, and length of stay. Secondary endpoints included BF in relation to the pathologic diagnosis of the resection specimens, time from surgery to detection of BF, and BF management.

### Statistical analysis

For statistical analysis, SPSS version 26 (SPSS, Inc., Chicago, Illinois, USA) for Windows (Microsoft Corporation, Redmond, Washington, USA) was used. Continuous variables were expressed as mean with standard deviation or median with range and were compared by using the independent-samples *t* test. Categorical variables were expressed as percentages and were compared by using Pearson's chi-square test with Yates' correction. Univariate and multivariate analysis using a binary logistic regression model was used to identify independent risk factors.

The study is a descriptive study. The principles stated in the Declaration of Helsinki have been recognized and followed. The use of register data followed the General Data Protection Regulation of the European Union and was approved by the Danish Data Protection Agency (RH -2015-07, nr. 03616), and patients' consent was obtained.

## Results

The majority of the patients were operated for a malignant tumor (Table 1). BF was, on average, detected on POD 5 (range POD 1–17). A total of 38 patients (6.7%) developed grade B and C BF (Table 1). BF and POPF occurred concomitantly in six patients, and the site of BF was at the site of the hepaticojejunostomy. Neither gender nor BMI was associated with the development of BF (*p* = 0.610 and 0.210), nor was ASA score (*p* = 0.080).

**Table 1.** Occurrence of Biliary Fistula and Baseline Characteristics

		BF		p
		Yes	No	
BF (incl. A), *n* (%)	49 (8.7)			
BF (excl. A), *n* (%)	38 (6.7)			
Grade A	11 (22)			
Grade B	32 (65)			
Grade C	6 (12)			
POPF (Grade B and C)		6 (12.5)	32	0.108
Gender, *n* (%)				0.610
Total	552			
Male	283 (51.3)	21 (7.4)	262	
Female	269 (48.7)	17 (6.3)	252	
Age, median (range)	69 years (16–90)	69 years (24–80)	69 years (16–90)	0.050
BMI, median (range)	24.5 (12.2–48.9)	24.6 (16.7–46.3)	24.5 (12.2–48.9)	0.210
ASA score, *n* (%)				
1	117 (21.2)	5 (4.3)	112	0.209
2	356 (64.5)	24 (6.7)	332	0.859
3	78 (14.1)	9 (11.5)	69	0.080
4	1 (0.2)	0 (0)	1	0.786

ASA, American Society of Anesthesiologists; BF, biliary fistula; BMI, body mass index; POPF, postoperative pancreatic fistulas.

However, BF was associated with the patient's age (*p* = 0.050) with an odds ratio of 0.973, and 72% of patients with BF had a small calibrated bile duct. BF was most common in patients operated for neuroendocrine tumors (16.0%) followed by patients with metastases to the pancreas from a primary tumor in other organs (11.1%). Patients with ductal adenocarcinomas had significantly fewer cases of BF (*p* = 0.006) (Table 2). When dividing the pathological diagnoses of the resection specimen into bile duct obstructing (pancreatic ductal adenocarcinoma, cholangiocarcinoma, and ampullary adenocarcinoma) and non-obstructing cases, there was a significant association between non-obstructive pathology and BF (*p* = 0.008).

**Table 2.** Histological Diagnoses and Overall Mortality

		BF		p
		Yes	No	
Overall 30-day mortality, *n* (%)	7 (1.3)	1 (14.3)	6	0.385
Overall 90-day mortality, *n* (%)	12 (3.4)	3 (25.0)	9	0.140
Histology, *n* (%)				0.008
Malignant	441 (79.9)	24 (5.4)	417	
Ductal adenocarcinoma	217 (39.3)	7 (3.2)	210	0.006
Periampullary adenocarcinoma	81 (14.7)	4 (4.9)	77	0.454
Duodenal adenocarcinoma	38 (6.9)	4 (10.5)	34	0.358
Other malignancies	49 (8.9)	2 (5.3)	47	0.417
Neuroendocrine tumor	25 (4.5)	4 (16.0)	21	0.065
Cholangiocarcinoma	21 (3.8)	2 (9.5)	19	0.626
Metastasis	9 (1.6)	1 (11.1)	8	0.614
Non-malignant	111 (20.1)	14 (12.6)	97	
Other pathology	63 (11.4)	8 (12.7)	55	0.053
IPMN	50 (9.1)	6 (12.0)	44	0.134

IPMN, intraductal papillary mucinous neoplasm.

Neither reconstruction techniques of the pancreaticojejunostomy (*p* = 0.458) nor vascular anatomy (*p* = 0.837) were associated with the development of BF.

Multivariate analysis of the identified risk factors did not yield any independent significant risk factors.

The average length of hospital stay after BF was 28 days compared with 17 (range 5–390) days for patients without a BF. The overall 30-day mortality was 1.3% (seven patients). One patient died of septicemia with multi-organ dysfunction syndrome and pneumonia caused by biliary peritonitis. The other six patients died due to complications directly related to PD, including intraabdominal hemorrhage, gastrointestinal bleeding, perforation of the colon, and acute respiratory distress syndrome.

The 90-day mortality was 3.4% (12 patients). Three of these patients (25%) had BF; however, the cause of death was unrelated to BF. These three patients suffered from systemic diseases, which had either exacerbated or acquired postoperative complications unassociated with BF. Table 3 provides a complete overview of the postoperative course in patients with BF.

**Table 3.** Postoperative Course in Patients with Detected Biliary Fistulas

BF, *n*	
Total BF (Grade B and C)	38
Postoperative course	
Length of stay (days)	32 (8–126)
Administration of antibiotics, *n*	38
Admission in an ICU, *n*	5
Placement of postoperative abdominal drain, *n*	18
Biliary drainage through PTC, *n*	37
Surgical site infection, *n*	7
Thromboembolisms, *n*	2
Reoperation, *n*	5
BF	2
Ischemia of the jejunum segment with anastomoses	1
Necrosis of the remaining pancreas	1
POPF	1
Intraabdominal abscess	11
POPF	6
Other complications	14
30-day mortality, *n*	1
Septic shock due to BF	1
90-day mortality, *n*	3
Unknown cause (death out of hospital)	1
Septic shock	2

ICU, intensive care unit; PTC, percutaneous transhepatic cholangiography.

Biliary drainage through PTC was performed in 37 of 38 patients, confirming a biliary leak from the hepaticojejunostomy before drain placement, and ultrasonographically guided intraabdominal drainage was performed in 18 patients after a CT scan revealed intraabdominal fluid collections. The patient with BF without biliary drainage was re-operated on POD 12 after the onset of acute septicemia. In total, five patients were re-operated, of whom two were due to BF, and five patients were transferred to the ICU (Table 2).

The two patients operated for BF were due a complete dehiscence of the anastomosis caused by a surgical technical issue and the other due to blow-out of the jejunal segment caused by an obstructing adhesion at the space in the mesentery of the transverse colon, through which the jejunal segment was passed retrocolically.

## Discussion

The incidence of BF was low, but almost all patients with BF had complications leading to an extended hospital admission of almost one month or more after the primary operation. A common cause of prolonged hospital stays for patients with and without BF was nutritional status and performance status, which required prolonged hospital stay to recover lost weight and physical strength. BF did not significantly increase 30-day mortality, as was also the case for 90-day mortality, where the causes of death were unrelated to BF, which in all three cases was sufficiently treated.

Among the assessed risk factors, the pathological diagnoses of the specimen and the size of the bile duct were associated with the risk of BF development. Patients with nonmalignant diagnoses had a greater risk of BF. More than 70% of patients with BF had a non-dilated CHD, defined radiologically or intraoperatively. Likewise, among malignant diagnoses, there were significantly more cases of BF in patients with malignancies not obstructing the CBD. As identified in previous literature, small duct diameter is among the main risk factors for BF.

Malignant obstruction of the bile duct is primarily caused by pancreatic ductal adenocarcinomas, cholangiocarcinomas, and ampullary adenocarcinoma.^14^ Neuroendocrine tumors, metastases from a primary tumor located elsewhere, some duodenal adenocarcinomas, intraductal papillary mucinous neoplasms, and other nonmalignant diagnoses are rarely a cause of obstruction of the CBD and so the associated fragile ducts with small duct diameter could explain the higher incidence of BF.^4^

Apart from the texture and calibration of the bile duct, the resection level of the bile duct and the vascularity seem to be of importance. To get optimal vascularization, the resection of the bile duct should be at the hepatic duct level, superior to the cystic duct but below the confluence. Ischemic cholangiopathy is commonly caused by skeletonization, division, and anastomosis with the CBD and ligation of the hepatic arteries. The hepatic arteries are the main suppliers of blood to the peribiliary vascular plexus supplying the CHD, whereas the right and left hepatic ducts are mainly vascularized by vessels from the hilar plate.^15^

The inferior part of the CBD is mainly supplied by the gastroduodenal artery, which is ligated during the operation.^6,16^ This leaves the remaining part of the CBD with lower vascularity than the hepatic duct, and so, an anastomosis to the CBD increases the risk of ischemic cholangiopathy and BF.^17^

In the effort to prevent BF, clinical setups and trials regarding various reconstruction techniques of the hepaticojejunostomy, preoperative and intraoperative percutaneous biliary drainage, transcystic drainage, and intraoperative T-tube placement have been attempted with little success.^4,7,18^ The development of BF seems multifactorial, especially late BF. Therefore, reducing the incidence of BF also seems multifactorial.

Early BF, detected before POD 3, are usually a result of technical failure, either due to surgeon inexperience, separation between sutures, ischemia due to placement of the anastomosis distally on the hepatic duct, microvascular ischemia due to a close distance between sutures, and tears in the duct due to traction from the sutures.^19^ Late BF is usually secondary to POPF, systemic stress, or localized fluid accumulations.^20^ To avoid early BF, a proper surgical technique with consideration of the texture, diameter of the hepatic duct, and the other earlier mentioned risk factors seems paramount.

Likewise, considering patient-specific risk factors and applying goal-directed therapy to reduce these risk factors could also help reduce the risk of BF. Among previous studies, old age, low levels of serum albumin, high BMI, combined liver resection and PD, and preoperative biliary drainage have been associated with a risk of BF.^1^ Even with a perfect surgical technique, patients will still be at risk of late BF. So, one should also consider methods of early detection and aggressive treatment, to avoid the complications related to BF.

Intraoperative placement of surgical drain aided in the detection of early BF, and these patients had a milder postoperative course. Insertion of a surgical drain to the hepaticojejunostomy and pancreaticojejunostomy is still a topic of discussion, as it may lead to BF as well as other morbidities.^1^

Recent reviews and international guidelines recommend selective placement of intraabdominal drains only if the operation was complicated and advocate early removal to avoid BF and increased morbidity.^21–23^ However, there is still no definitive consensus on the placement and positioning of an intraabdominal drain after PD, and large, well-established trials have yet to be completed. At our center, we routinely place an intraoperative drain, as we consider this a help to detect the early hepaticojejunostomy leaks, find POPF, and avoid larger fluid accumulations.

Similar to the placement of surgical drains, preoperative biliary drainage seems a topic of discussion. According to the European Society of Medical Oncology (ESMO) guidelines for the treatment of pancreatic cancer, preoperative biliary drainage should only be performed in cases of obstructive cholangitis and delayed surgery as drainage increases the risk of postoperative complications.^24,25^ However, recent studies show comparable complication rates by early biliary drainage compared with no drainage.^26^

As biliary obstruction increases the risk of cholangitis and transient or permanent kidney failure due to highly elevated bilirubin leading to toxic nephropathy,^27^ obstruction should be relieved by endoscopic placement of a biliary stent preferably, or external drainage by PTC, before complications occur that may postpone surgery.

The most apparent limitation of our study is the retrospective design, though we have used data from a prospective pancreatic surgical registry with predefined characteristics and outcomes. Another limitation is the lack of standardized documentation of certain known risk factors for BF, including intraoperative blood loss, specified bile duct diameter at the transection site, and texture, especially in the procedures in the beginning of the assessed period.

A major strength of our study is the large sample size from a single high-volume center with strict guidelines in postoperative care and in case of complications. The study included patients operated on during recent years, which makes it a good reflection of current clinical practice.

## Conclusion

The incidence of BF is low, though BF is associated with an increase in complications, prolonged hospital admissions, and transfer to ICU. Patients with small size of the extrahepatic bile duct run the greatest risk of postoperative BF. Proper surgical technique with consideration of the texture, diameter of the hepatic duct, and applying goal-directed therapy to patient-specific risk factors could help reduce the risk of BF.

## Abbreviations Used

ASA: American Society of Anesthesiologists
BF: biliary fistula
BMI: body mass index
CBD: common bile duct
CHD: common hepatic duct
CT: computed tomography
ICU: intensive care unit
IPMN: intraductal papillary mucinous neoplasm
OR: odds ratio
PD: pancreaticoduodenectomy
POD: postoperative day
POPF: postoperative pancreatic fistulas
PTC: percutaneous transhepatic cholangiography
SD: standard deviation
